# Quantized conductance coincides with state instability and excess noise in tantalum oxide memristors

**DOI:** 10.1038/ncomms11142

**Published:** 2016-04-04

**Authors:** Wei Yi, Sergey E. Savel'ev, Gilberto Medeiros-Ribeiro, Feng Miao, M.-X. Zhang, J. Joshua Yang, Alexander M. Bratkovsky, R. Stanley Williams

**Affiliations:** 1 Hewlett-Packard Laboratories, Palo Alto, California 94304, USA; 2 HRL Laboratories, LLC, Malibu, California 90265, USA; 3 Department of Physics, Loughborough University, Loughborough LE11 3TU, UK; 4 Departamento de Física, UFMG, PO Box 702, Belo Horizonte, 30123-970, Brazil; 5 National Laboratory of Solid State Microstructures, School of Physics, Collaborative Innovation Center of Advanced Microstructures, Nanjing University, Nanjing 210093, China; 6 Department of Electrical and Computer Engineering, University of Massachusetts, Amherst, Massachusetts 01003, USA; 7 Department of Physics, University of California, Davis, California 95616, USA; 8 P. L. Kapitza Institute for Physical Problems, 2 Kosygina Str., 119334 Moscow, Russia

## Abstract

Tantalum oxide memristors can switch continuously from a low-conductance semiconducting to a high-conductance metallic state. At the boundary between these two regimes are quantized conductance states, which indicate the formation of a point contact within the oxide characterized by multistable conductance fluctuations and enlarged electronic noise. Here, we observe diverse conductance-dependent noise spectra, including a transition from 1/*f*^2^ (activated transport) to 1/*f* (flicker noise) as a function of the frequency *f*, and a large peak in the noise amplitude at the conductance quantum *G*_Q_=2*e*^2^/*h*, in contrast to suppressed noise at the conductance quantum observed in other systems. We model the stochastic behaviour near the point contact regime using Molecular Dynamics–Langevin simulations and understand the observed frequency-dependent noise behaviour in terms of thermally activated atomic-scale fluctuations that make and break a quantum conductance channel. These results provide insights into switching mechanisms and guidance to device operating ranges for different applications.

## Abstract (web summary)

Oxide memristors exhibit noise in excess of 2–4 orders of magnitude above the baseline at quantized conductance states. Here, the authors measure anomalous electrical noise at these states in tantalum oxide memristors and relate it to thermally-activated atomic fluctuations by numerical simulations.

## Graphical abstract

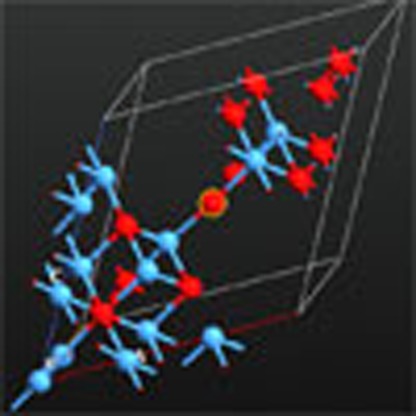

TaO_*x*_-based metal-oxide-metal devices exhibit voltage-induced resistance switching that represents a physical realization of the memristor electronic circuit element model^1^,^2^,^3^. They are interesting for device and circuit applications^4^,^5^,^6^,^7^,^8^,^9^,^10^,^11^,^12^ because of their high ON-to-OFF switching endurance^13^,^14^, sub-nanosecond switching times^15^,^16^, and picoJoule switching energies^17^. At the same time, spectromicroscopic studies^18^, transmission electron microscope analysis^19^ and temperature-dependent transport measurements^20^ have shown that the resistance switching mechanism in the TaO_*x*_ system is significantly different from that in TiO_2_-based memristors^21^,^22^,^23^,^24^. In particular, the primary state variable that determines the electronic transport mechanism in TaO_*x*_ memristors has been identified to be the oxygen content in a TaO_*x*_ channel running through the film^19^,^20^. Other state variables, such as the internal temperature and the diameter of the conduction channel^25^, may also influence the resistance. At low oxygen content (∼20% O dissolved in Ta), the channel displays metallic conductivity, whereas at higher oxygen content (larger than 50%, a suboxide, or reduced oxide) the channel can display a range of conduction behaviours, including activated semiconducting transport, barrier tunneling and hopping, all characteristic of a Fermi glass^26^, which is reminiscent of the behaviour described by the Mooij rule for disordered transition metals^27^.

An intermediate regime of electronic transport is characterized by quantized conduction, typically observed in mechanical break junctions^28^,^29^,^30^ but also in metal–insulator–metal resistive switches^31^,^32^,^33^,^34^. Here we focus primarily on this regime, when the resistance of the memristor is in the 3 to 15 kΩ range (or in the range of a few conductance quanta *G*_Q_=2*e*^2^/*h*=77.5 μS=(12.9 kΩ)^−1^). We observe excess noise at conductance quanta for different samples and temperatures, several orders of magnitude above the baseline. The origin of excess noise is explained in terms of atomic instabilities at the contact. These results provide insight into switching mechanisms in memristors, as well as define regions for device operation for different applications.

## Results

### Current voltage characteristics near quantum point contacts

**Figure 1:**
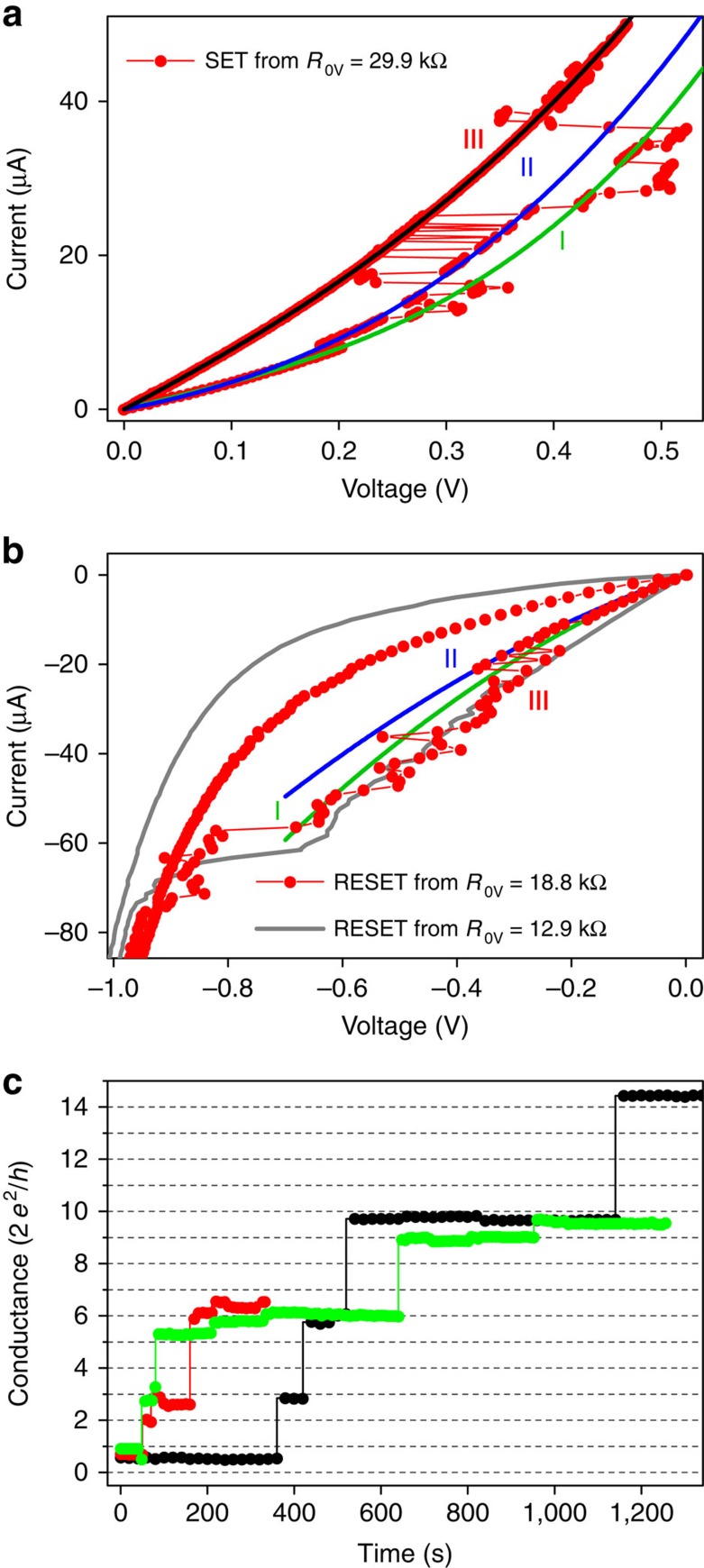
*I*–*V* and stress characterizations of TaO_*x*_ devices. Multistate resistance fluctuations resembling telegraph noise are evident for nominal (**a**) SET and (**b**) RESET sweeps. Conductance fluctuations (red, experimental data) were observed among a set of nonlinear I-V curves (indicated by colored lines: initial states I, intermediate states II, quantum resistance states III) for both positive and negative current ramps applied to TaO_*x*_ memristors when one of the states had a zero-bias resistance near the resistance quantum *R*_Q_ (*h*/2*e*^2^=12.9 kΩ). (**a**) SET operation from *R*_0V_=29.9 kΩ. (**b**) RESET operation from *R*_0V_=12.9 kΩ (grey) and *R*_0V_=18.8 kΩ (red with solid circles). (**c**) Temporal evolution of zero-bias conductance states in TaO_*x*_ memristors during low-voltage stress tests that has been performed by applying a pulse with 150 mV amplitude and 20-μs duration followed by reading the device conductance at 50 mV, with the sequence repeated at a time interval of 20 s. The device conductance jumps between values corresponding to an integer times the conductance quantum (2*e*^2^/*h*). Three traces with different colours (red, black and green) represent three separate tests on the same device.

Our devices were sputter-deposited TaO_*x*_ memristors with a bottom (grounded) Pt electrode and a top Ta electrode. We measured current controlled quasi-static current–voltage (*I*–*V*) sweeps, conductance as a function of time for constant applied voltage, and frequency-dependent noise spectra (see details in Methods, Supplementary Fig. 1 and Supplementary Note 1). The *I*–*V* characteristics in Fig. 1a,b for SET and RESET operations display conspicuous telegraph-type dynamical fluctuations when the conductance is lower or equal to *G*_Q_ (see also Supplementary Note 2). Thus, the device states near *G*_Q_ are highly unstable, with the conductance hopping back and forth among several different and apparently discrete states as the current is ramped. The dynamical and stochastic nature of switching in this regime is shown in Fig. 1c, which displays the conductance versus time of a device held at a constant voltage of 50 mV. The conductance exhibited jumps at apparently random time intervals to more conductive states at essentially integer multiples of *G*_Q_.

**Figure 2:**
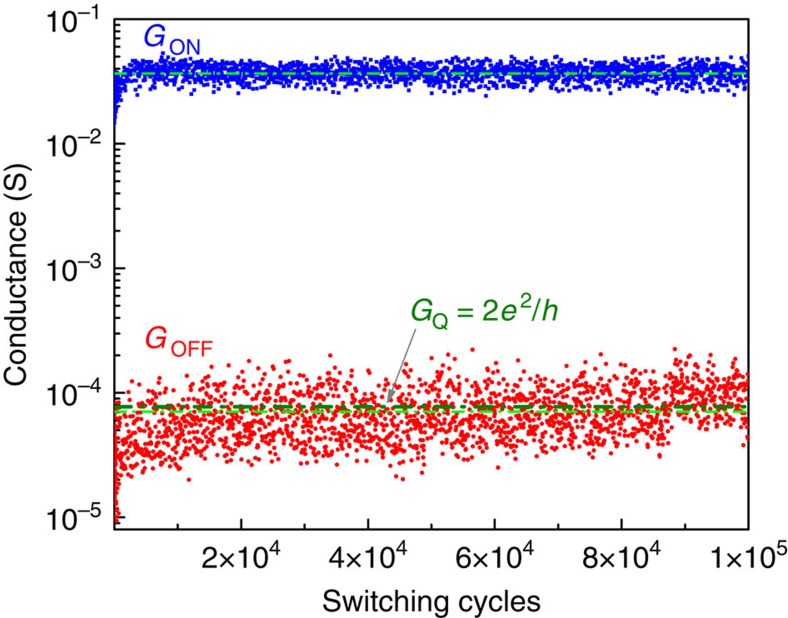
Conductance behaviour in consecutive ON/OFF switching events. Conductance switching data of a TaO_*x*_ memristor for 100,000 switching cycles showing larger variance in the regime close to a quantum of conductance. The solid symbols represent conductance data of ON (blue) and OFF (red) states and the green dash dot lines represents the corresponding average values during the 100,000 cycles. The mean conductance value and s.d. are <*G*_ON_>=3.67 × 10^−2^ S and *σ*_ON_=5.7 × 10^−3^ S for ON states, and are <*G*_OFF_>=7.06 × 10^−5^ S and *σ*_OFF_=3.38 × 10^−3^ S for OFF states. Thick olive dash dot line highlights the conductance quantum (2*e*^2^/*h*).

In Fig. 2 we illustrate how these fluctuations affect resistance switching by cycling a device between two different states over 100,000 times. The average conductance <*G*> showed a significantly enhanced variance when we attempted to reset the device to a nominal value of *G*_Q_.

### Noise spectra

**Figure 3:**
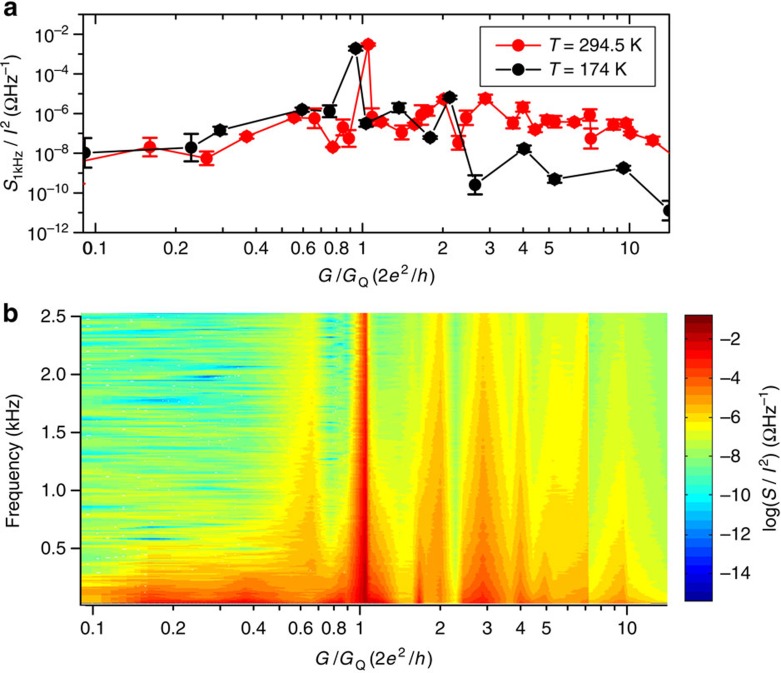
Conductance dependence of normalized noise spectral density. The normalized noise spectral density *S/I*^2^ for a TaO_*x*_ memristor are plotted as a function of *G*_Q_-normalized zero-bias conductance. (**a**) *S/I*^2^ at 1kHz for two temperatures, *T*=295 K and 174 K (**b**) two-dimensional colour plot showing *S/I*^2^ as a function of conductance *G*/*G*_Q_ and frequency *f* at *T*=295 K. The continuous scale on the colour plot was obtained by linear interpolation between measured data points. This representation clearly reveals the markedly increased noise near the first conductance quantum *G*_Q_ and the tails of the noise distribution as a function of frequency. In addition, increased noise above the ambient trend can be seen for several other integer multiples of *G*_Q_. The error bars in **a** are s.d. calculated from evaluating *S/I*^2^ at different sourced currents (Supplementary Note 1). Small temperature dependence of noise at around *G*=*G*_Q_ and at smaller conductance values (corresponding to a tunneling regime) confirms the present picture of local heating as the main source of noise (see text).

Figure 3 shows that the current noise spectral density in a device can be a few orders of magnitude larger than the background values for conductance values close to small integer numbers times *G*_Q_. The detailed conductance and frequency dependence of the electrical noise in a TaO_*x*_ memristor is exhibited in Fig. 3. The upper panel shows the noise normalized by the square of the current at 1 kHz for room temperature and 174 K. *S* is the noise power spectral density in units of WHz^−1^, while *S/I*^2^=*S*_*R*_ gives the resistance noise spectral density in units of ΩHz^−1^. The lower panel shows a 2D chart of the noise amplitude plotted as a function of device conductance and frequency for room temperature data. In both plots, there is a strong peak in the noise at *G*_Q_, which rises three orders of magnitude above the baseline, and several minor peaks that appear primarily at integer values of *G*_Q_. These general features were observed for similarly prepared samples, but the peak intensities and the presence or absence of a peak at particular integer multiples of *G*_Q_ (*nG*_Q_) varied from sample to sample (see Supplementary Fig. 2).

**Figure 4:**
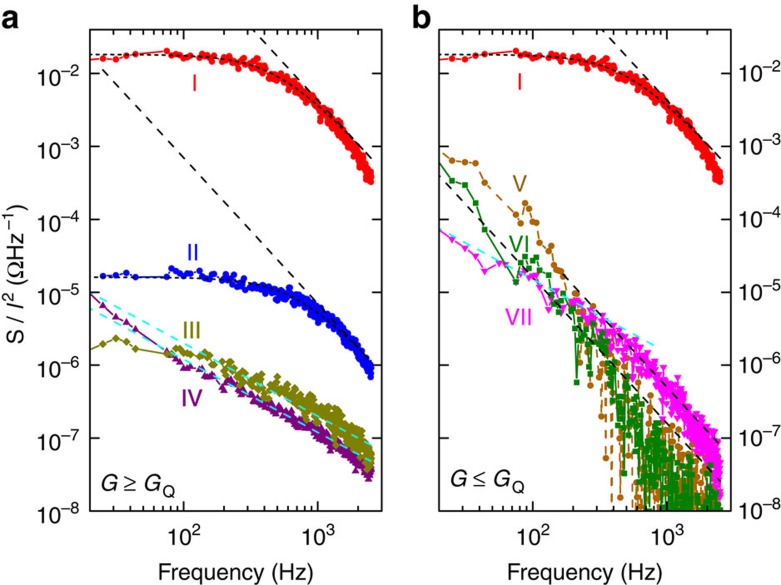
Frequency dependence of normalized noise spectral density. The normalized noise spectral density *S/I*^2^ are plotted as a function of frequency *f* for seven different conductance states of a TaO_*x*_ memristor. (**a**) states with *G*≥*G*_Q_. (**b**) states with *G*≤*G*_Q_. The general trends are that the lower conductance states have larger normalized noise amplitudes, metallic states (for example, *R*(III)=8.24 kΩ and *R*(IV)=1.28 kΩ) show a 1/*f* functional dependence (cyan dashed lines), while semiconducting states with higher resistance follow a 1/*f*^2^ law (black dashed lines). The spectrum of a system close to one resistance quantum of *R*(I)=12.3 kΩ is two orders of magnitude higher in amplitude at high frequency than the others represented in the plot. Also shown for the device conductance states at multiples of *G*_Q_ the characteristic Lorentzian spectrum signature of atomic fluctuators (short dashed lines as Lorentzian fits for *R*(I)=12.3 kΩ and *R*(II)=6.4 kΩ). Note peculiar crossover between 1/*f* law at lower frequencies to 1/*f*^2^ law at higher frequencies in *R*(VII)=23.3 kΩ sample that matches the simulation in Supplementary Fig. 5c. More resistive *R*(VI)=49.4 kΩ and *R*(V)=80.6 kΩ samples mostly follow 1/*f*^2^ law.

Figure 4 shows the measured frequency-dependent noise spectra over a wide range of conductance states. The frequency (*f*) dependence of the noise was 1/*f* for the high-conductance states and 1/*f*^2^ for the low-conductance states. However, in the quantized conductance regime, the noise amplitude was significantly above the background trend for *G*=*nG*_Q_ (*n*=1 and 2). At *G*=*G*_Q_, the noise displayed a 1/*f*^2^ dependence at high frequencies but flattened out at lower frequencies.

There is a vast literature on quantized conductance in Quantum Point Contacts (QPC) in mechanical break junctions (see, for example, refs 28, 29, 30 and references therein) that show similar behaviour to Fig. 1c. A single- or few-atom constriction can support few-electron eigenmodes that apparently suffer little back-scattering. Similar conductance steps have been observed in several thin film systems, for example, Ag_2_S (ref. 31), AgI (ref. 32) and more recently in Ag/GeS_2_/W (ref. 33), Ta_2_O_5_ (ref. 35), and Nb/ZnO/Pt (ref. 36), thus demonstrating that QPCs can also exist in metallic systems embedded in insulating matrices.

A phenomenological model reported by Ielmini *et al*.^37^ describes telegraphic noise during the transition from insulating to metallic regime. Telegraphic noise can depend on both the conducting channel width^37^ as well as applied voltage^38^. However, enlarged noise behaviour at a QPC has not been revealed in any system to the best of our knowledge. The control experiment for noise in QPCs was performed in 2D electron gases in GaAs/AlGaAs heterojunctions^39^. For this pristine system, at integer numbers of conductance quanta, noise was actually suppressed. In a later study on clean Au mechanical break junctions^40^, a similar behaviour was observed. More recently, shot noise was evaluated in mechanical break junctions with molecules in the channel, and noise was also suppressed^41^. These findings contrast with the enhanced noise and its temperature independence reported here. We attribute the enlarged noise to thermally activated atom motion in the conductance channel of the memristive system.

Resistive switches are excellent controlled environments to investigate QPCs and the corresponding noise behaviour, since the lifetime of a particular resistance state can be sufficiently long for a detailed transport analysis. The conductance state instability and enlarged noise at *G*=*nG*_Q_ values can be explained in terms of a thermally fluctuating point contact in the oxide film of the memristor. In Fig. 4, the noise frequency dependence at *nG*_Q_ exhibits a Lorentzian behaviour 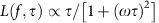, which is a signature of a single fluctuator, that is, potentially a single-atom jumping inside the point contact with a particular frequency cut-off (typically several hundred Hz, refs 42, 43). This fluctuator alternates among different electronic eigenstates with essentially the same conductance, since the noise peaks are relatively narrow and nearly centred at values of *nG*_Q_. Qualitatively, when a conductance channel first forms in the oxide that just bridges the electrodes of the memristor, a hot spot forms at the narrowest part of the channel, which can be an atomic-scale point contact through which most of the current in the device flows. This produces high local Joule heating, which in turn causes atomic fluctuations and severe electronic noise because of the disruption of the conductance channel. The noise peaks at *nG*_Q_ with *n*=2, 3 and so on. may be caused by multiple point contacts in parallel or a single fluctuating contact that supports degenerate electron eigenstates^29^.

The existence of a peak in the noise at a discrete conductance can be explained by a simple local bond model of a random network of resistances *r*_*m*_={*r*,∞} (ref. 44, and see Supplementary Note 3 and Supplementary Figs 3 and 4). When the concentration of the conducting bonds decreases to the critical value for disconnecting a conducting channel between the electrodes, the electronic noise diverges because the fluctuations of the conductance are determined by a small number of bonds at the point contact^45^,^46^,


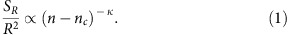


In a physical system, this divergence will be smoothed out by tunneling through the gap in the channel^46^,^47^ and by shunting around the gap through the surrounding matrix material (for example, the nearly stoichiometric oxide).

If there are several parallel conducting paths through the device, then the fluctuations of local conductances are uncorrelated and their contributions to the total conductance are averaged. We do not know exactly the microstructure of the growing embryonic conducting channels, but our observations are quite general to any geometry that includes point contacts. It is frequently assumed that they make a dendritic (or a stalagmite/stalactite) structure. In the case of the formation of a few parallel QPCs, the amount of local heating at each would obviously be lower than the single QPC case and may lead to a series of weaker noise peaks, since the amount of local heating in an ideal case for constant current would fall off with *n*^2^, *n* being the number of parallel channels.

## Discussion

We compare our theoretical expectations with the observed behaviour of the normalized noise spectra *S/I*^2^ for resistance states around *G*_Q_ shown in Fig. 4. We observe three distinct regimes: a metallic, a semiconducting or insulating one and an intermediate regime characterized by conductances of *nG*_Q_. The metallic state with *R*=1.28 kΩ exhibits 1/*f* noise, while the semiconducting state shows 1/*f*^2^ behaviour at high frequencies. In the 23 kΩ sample (close to 0.5 *G*_Q_), we observe the crossover from 1/*f* to 1/*f*^2^ reproduced by our Molecular Dynamics–Langevin (MD-L) model (see Supplementary Fig. 5), and more resistive samples follow the 1/*f*^2^ behaviour. The samples in the quantized conductance regime around 12 kΩ exhibit the largest noise power compared with samples outside of this regime, exhibiting a Lorentzian distribution with <*G*>=*G*_Q_. For <*G*>=2*G*_Q_, (*R*=6.4 kΩ), we observe a significantly lower power but also a Lorentzian behaviour. These results are in accord with discrete atomic fluctuators at the point contact, which is the predominant source of electronic noise level for the entire device.

Noise measurements can be a very sensitive probe of the internal dynamics of atomic-scale fluctuations in memristors and related devices. The observed multistable current–voltage characteristics and severe electronic noise in TaO_*x*_ memristors for quantized conductance states can be explained by a simple model of atomic thermal fluctuations that disrupt electronic eigenstates in a point contact of a conducting channel, and should be a general phenomenon in resistance switching based on atomic or ionic migration. Thus, although the possibility of having discrete conductance states in a device appears attractive, the inherent instability of the states can present a challenge for non-volatile memory applications^9^,^48^ near point-contact regime. In previous work we showed that one can operate in quiet windows in between conductance quanta by controlling the write current^20^ or using a feedback write circuit^49^. Nevertheless, there are applications where noise can be used as a resource. One example is stochastic resonance phenomena in signal processing, wherein the addition of white or 1/*f* noise can enhance the response of a nonlinear system to subthreshold signals^50^,^51^,^52^. This phenomenon suggests an important role for noise in information processing. In the brain, stochasticity in synaptic inputs can help in cognitive processes such as decision making and learning^53^,^54^. Another application is the use of noise for the realization of physical sources of random number generators^55^. Traditionally embodied in optical schemes (see, for example: http://www.idquantique.com) to harness quantum properties of light as the phenomenon generating randomness, small footprint memristor devices can present a competitive advantage in integrated circuits. Correlation tests are needed to further demonstrate the quality of the random data for cryptography and other applications such as stochastic computing^44^. These examples illustrate that beyond non-volatility, there are novel applications for memristors when it comes to exploring microscopic phenomena governing their behaviour, with noise as an asset.

## Methods

### TaO_*x*_ devices

The TaO_*x*_ films of the devices were sputtered from a tantalum oxide target (nominal composition to be Ta_2_O_5_) with an Ar gas pressure of about 3 mtorr. The device substrate was a commercial 200 nm SiO_2_/Si(001) wafer. The disc device stack consisted of (from bottom to up) 1 nm Ti blanket adhesion layer, 100–400 nm Pt blanket bottom electrode, 10 to 18 nm TaO_*x*_ blanket layer, and 100–400 nm Ta disc (50 to 200 μm diameter) top electrodes. Metallic Pt and Ti layers were deposited by electron-beam evaporation at ambient temperature and the metallic Ta contacts were DC sputtered at ambient temperature through shadow masks.

### Electrical measurements

Quasi-static sweeps. Current–voltage (*I*–*V*) curves and resistance measurements were performed in a standard four-point probe configuration using an Agilent B1500A parameter analyzer equipped with 4 source measure units (SMUs). The measured contact resistance of the top and bottom electrodes was less than 60 Ohm. A linear sweep mode with constant V or I ramp rates was used for V-driven and I-driven *I–V* curves, respectively. Fixed gains (ranges) were used for *V* (*I*) readings to avoid any disturbance due to gain (range) adjustment. For the setup with short integration time and autogain, the measurement interval time varied between a few and tens of milliseconds. The *I* ramp rate set by the sweep range, the SMU integration time, the SMU delay time, and the number of data points was on the order of 10^−2^ to 10^−5^ A s^−1^. The V ramp rate used was 0.1–10 V s^−1^.

## Additional information

**How to cite this article:** Yi, W. *et al*. Quantized conductance coincides with state instability and excess noise in tantalum oxide memristors. *Nat. Commun.* 7:11142 doi: 10.1038/ncomms11142 (2016).

## Supplementary Material

Supplementary Information Supplementary Figures 1-5, Supplementary Notes 1-3 and Supplementary References
