# Supplementary material for: Quantized conductance coincides with state instability and excess noise in tantalum oxide memristors
Source: Nat Commun. 2016 Apr 4;7:11142. doi: 10.1038/ncomms11142 (PMC4822004; doi:10.1038/ncomms11142)
Supplement: Supplementary Information — Supplementary Figures 1-5, Supplementary Notes 1-3 and Supplementary References [file ncomms11142-s1.pdf]

Supplementary Figure 1

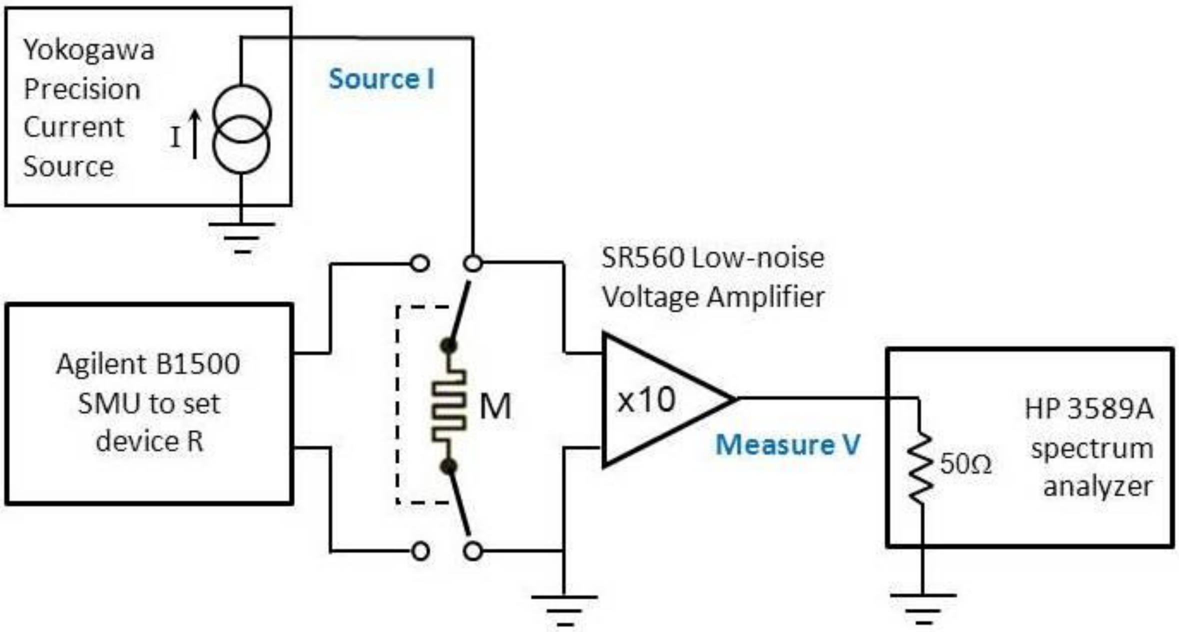

Supplementary Figure 1. Schematic of a setup to measure electrical noise (see text).

Supplementary Figure 2

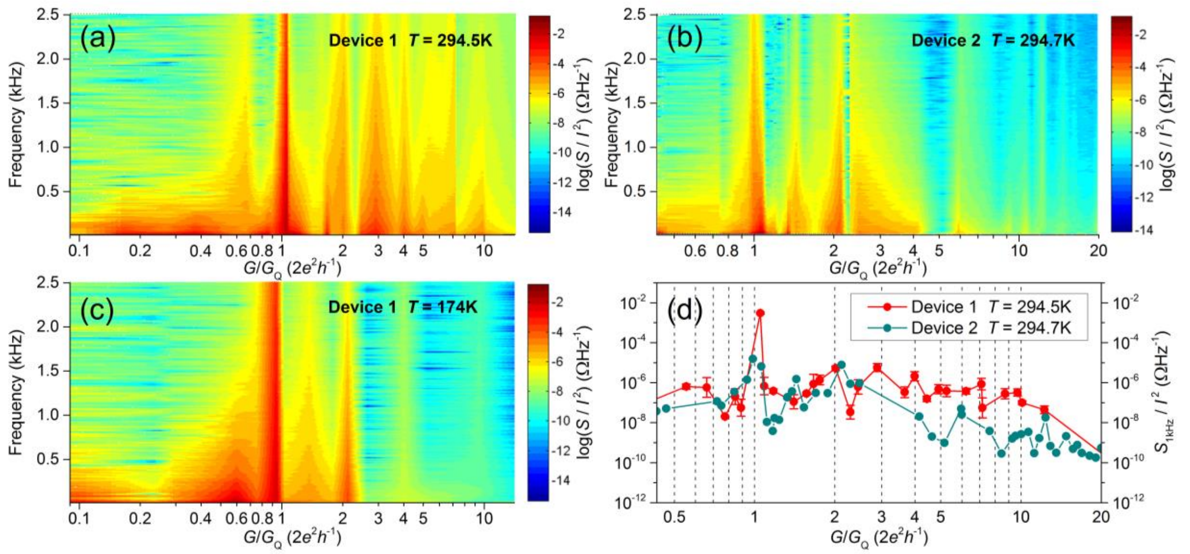

Supplementary Figure 2. (a,b) Spectral noise intensity maps Log(S/I^2) for devices 1 (discussed in the main text) and 2 (fabricated with the same nominal parameters as device 1), measured at room temperature. (a,c), temperature dependence of the spectral noise intensity for device 1. (d) The room-temperature normalized electronic noise power S_{1kHz}/I^2 for devices 1 and 2 versus their conductance. - The error bars are standard deviations calculated from evaluating S/I^2 at different sourced currents (see Supplementary Note 1).

Supplementary Figure 3

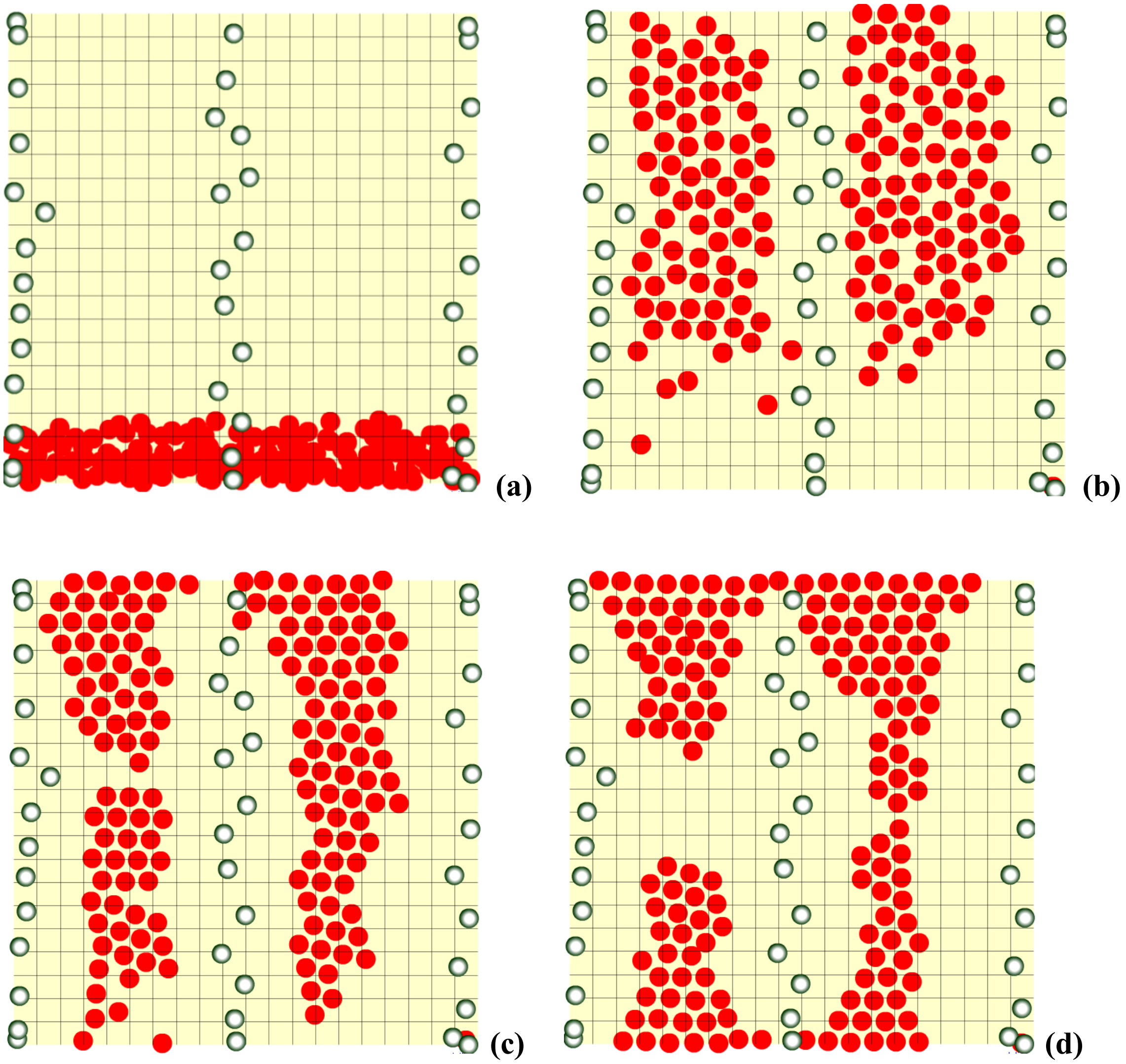

Supplementary Figure 3. Evolution of the vacancy distribution simulated using the model expressed by Eqs.(1)-(3) in a sample with three columnar defects and periodic boundary conditions on a lateral scale of $20r_c$. (a) Just before applying the switching pulse (all vacancies are located at the bottom of the simulated area); (b) Just after application of the switching pulse, which spreads the vacancies in the simulated memristors; (c) after a time that is three times longer than the pulse duration, we see a vacancy stalagmite growing in the right part of the sample; (d) after long time (ten times longer than the pulse duration), the point contact was formed in the right part of the sample, while the top and bottom vacancy clusters are disconnected in the left part of the sample, showing the stochastic nature of QPC formation.

Supplementary Figure 4

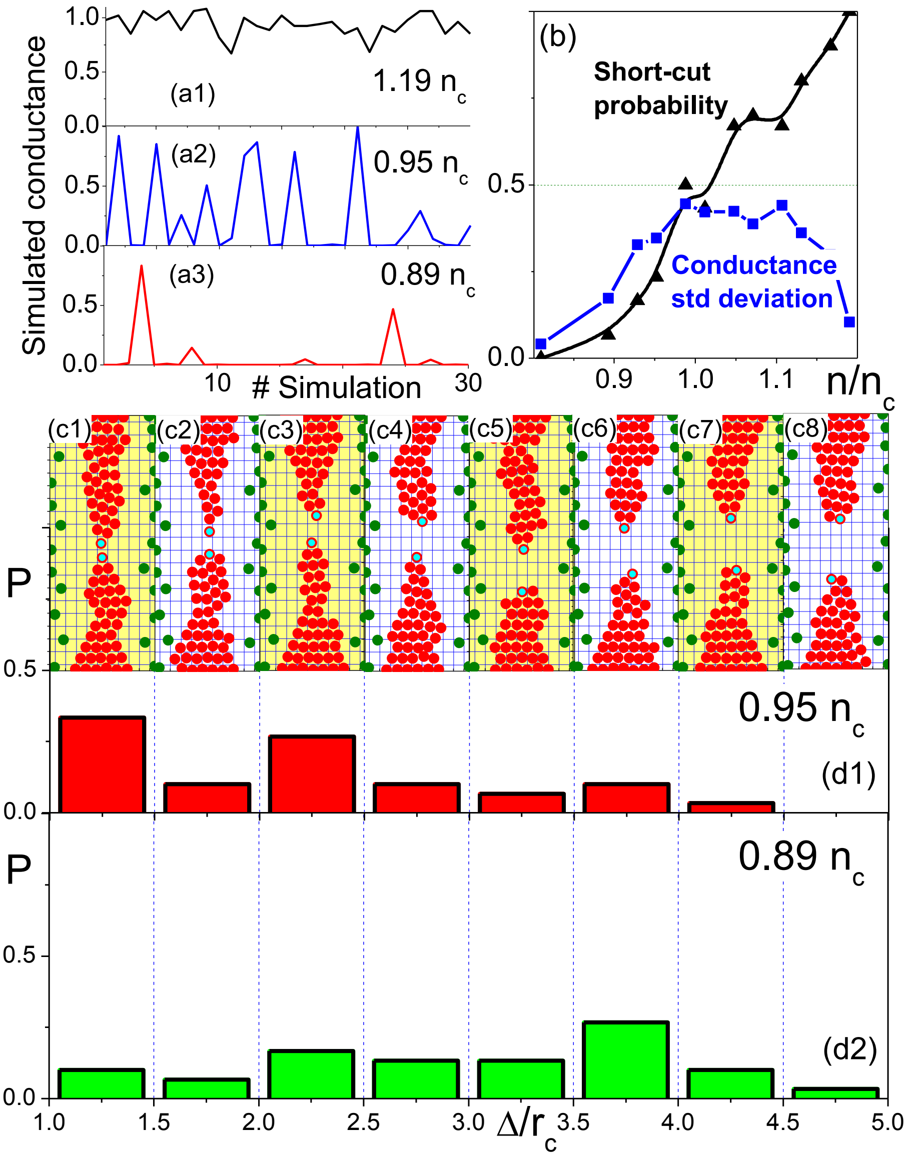

Supplementary Figure 4. Simulated stochastic making and breaking of the quantum point contact in a memristor with different vacancy concentrations n: (a1) n=1.19n_c is above the connecting threshold where contacts are very likely to form, (a2) n=0.95n_c is near the critical value n_c, where for about 50% of simulations an atomic point contact is formed, and (a3) n=0.89n_c is well below the threshold where the contact rarely forms. Panel (b) shows the standard deviation of G as a function of the vacancy concentration n, which illustrates the significant enhancement of noise around n=n_c. The probability of forming a contact steadily increases with vacancy concentration, as expected. Panels (c) and (d) illustrate the statistics (frequency histograms) of forming a system with a particular gap Δ/r_c (the horizontal axis) for n=0.95n_c (d1) and n=0.89n_c (d2) between metallic-like (vacancy) protrusions. The panel (c) shows typical atomic configurations with a contact formed for $\Delta/r_c \approx 1$ (c1,c2) and well separated for $\Delta/r_c \geq 3$ (c4-c8). The local geometry, power, and temperature during reconfiguration of the contact were calculated self-consistently to achieve these results.

Supplementary Figure 5

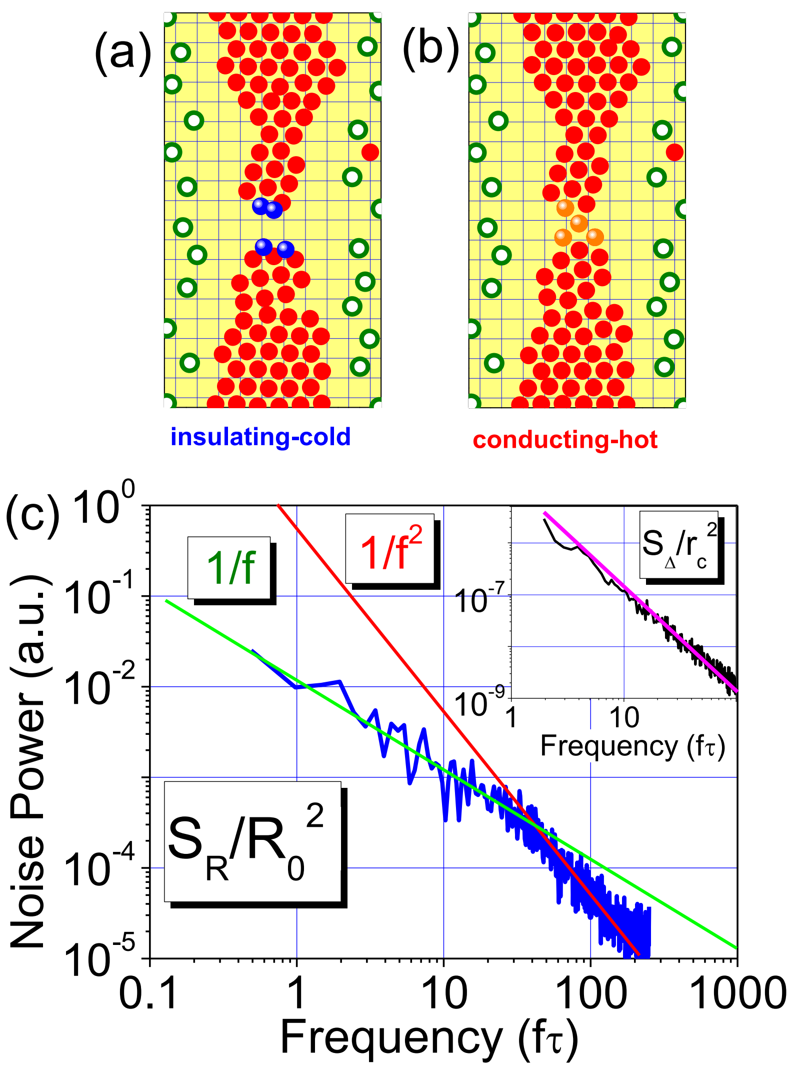

Supplementary Figure 5. Local atomic configurations occurring at different times during the simulations and some noise power spectra. (a) a discontinuous junction with a tunnel gap Δ, low conductance and lower local temperature; (b) a continuous Ohmic junction with relatively high conductance and higher local dissipated power and temperature. (c) Stochastic switching between (a) and (b), i.e. between open to closed junctions results in a peculiar $1/f$ to $1/f^2$ crossover due to the exponential dependence of the bond resistance on the gap Δ between the two conducting clusters, Eq.(3), while the noise of the gap itself is $1/f$ (c, inset). The local geometry, power, and temperature during the reconfiguration of the contact have been calculated self-consistently. Some samples with R at or somewhat below R_Q exhibit the noise spectrum flattening at low frequencies, typical of the noise determined by a single fluctuator, while at $R>R_Q$ there is a crossover between the two power-law regimes, $1/f^p$, from p=1 to p=2. This latter behavior is captured by the present simulations.

Supplementary Note 1: Experimental Procedure

Low-voltage Voltage stress measurements

Stress tests as shown in Supplementary Fig. 1(c) were conducted with a commercial Agilent 4156C parameter analyzer equipped with 4 source measure units (SMUs). The stress test was performed by applying a pulse of 0.15V (20μs) followed by reading the device resistance at 50mV, and repeat the sequence at a time interval of 20s.

Noise measurements

A very high output impedance Yokogawa 7651 DC source supplied a constant DC current for noise measurements. The source current was varied from 0.2μA, 0.5μA, 1μA, 2μA, ..., up to 10μA near G_Q, an order of magnitude higher for low resistance states (*i.e.*, less than approximately 5-10kΩ), and an order of magnitude lower for higher resistance states (i.e., more than approximately 10-20kΩ) . The noise voltage across the sample was amplified by a Stanford Research System SR560 low-noise voltage amplifier with an input impedance of 100MΩ, and the output signals were analyzed by an HP 3589A spectrum analyzer. From the various sourced currents, an average value was obtained and error bars were derived from the standard deviation of the obtained data. The schematic setup is shown in Supplementary Fig.1. In all the measurements, the bottom electrode (Pt) was grounded and the current was fed into the top electrode (Ta). An Agilent B1500A parameter analyzer was used to switch the device to different conductance states in between noise measurements. The bandwidth of SR560 amplifier is 1 MHz, and a fixed gain of 10x was used for all the device conductance states. The HP 3589A spectrum analyzer was set with a frequency range of 10-2500 Hz with 100 times spectrum averaging. The background noise level of the measurement setup was collected by supplying zero current to the device and it was subtracted from the measured noise spectrum to obtain the actual noise level originated from the device. This background noise level was found to be temperature- and device-independent, and its power spectral density (PSD) scales with the square of device resistance at low frequencies. The zero-resistance intercept (the noise floor) was found to be $0.3pW.Hz^{-1}$. This instrument noise floor is negligibly small in all noise measurements.

Supplementary Note 2: Current-voltage characteristics

Conventional I-V characteristics are taken by sweeping the voltage and measuring the current. Although such a procedure is quite adequate for a range of loads, due to the series resistance and finite input resistance of the current-to-voltage amplifier, the voltage drop across the device and the current through the device are not constant. As such, one can only catch the first (major) switching event while the subsequent switching events are obscured as the resistance state continuously evolves. We have instead utilized a *constant current* source with very high output impedance, and measured the voltage drop across the device. Although generally speaking the two schemes should produce similar results for typical device impedances (around 1kΩ-20kΩ), we found that under constant current stimuli the switching and fluctuations were discrete, and in between switching events the device would lock to a particular I-V locus, i.e. a particular non-volatile resistance state. The resistance noise is measured at selected resistance states as voltage fluctuations across the device under constant current. The current is chosen in such a way that it is a minor perturbation and does not significantly change the resistance during the experiment. The signal is amplified by a high bandwidth voltage amplifier and further analyzed by a spectrum analyzer.

We measured current-controlled I-V curves for SET and RESET operations [see Figs 1(a), 1(b) in the main text]. For all cases, the device conductance was near the quantum of conductance G_Q =(12.9 kΩ)$^{-1}$ (for the SET operation, the *final* conductance was close to G_Q, for RESET the *initial* conductance was close to G_Q.).

Supplementary Note 3: Theory and Modeling

Resistance noise

To gain more insight into the noise peak at G=G_Q, one can rewrite the total dissipated power in the junction RI^2, where the I is the total (fixed in experiment) current through the junction, via local electric fields E_α and conductivities $\sigma_{\alpha\beta}$ in a standard manner [1]:

$$I^2R = \int dV\, j_\alpha(r)E_\alpha(r) = \int dV\, E_\alpha(r)\sigma_{\alpha\beta}(r)E_\beta(r) \quad (1)$$

In oxide junctions, the local fields are extremely inhomogeneous, especially near G=G_Q, where most of the voltage drops across just a few-atom bottleneck that determines the total resistance R. To gain more insight into the importance of these hot spots, we can formally discretize the lattice into a random network of bond resistances with $r_m = \{r, \infty\}$, m=1, 2, ... The resistance

fluctuation is then $\delta R(t) = \sum_m i_m^2\, \delta r_m(t)$, where $i_m = I_m/I$ is the relative current through the bond between entities m, which may be vacancies, and the correlation function of the bond resistances can be expressed through the spectral density $s(f)$ of resistance fluctuations via $2\langle \delta r_m(t_1)\delta r_n(t_2)\rangle_\omega = s(f)r^2\delta_{mn}$. This relations allow one to express the relative resistance noise in the junction as[1]

$$\frac{S_R(f)}{R^2} = s(f)\frac{\sum_m i_m^4}{\left(\sum_m i_m^2\right)^2} \tag{2}$$

which diverges in the above classical binary bond resistance case near the percolation threshold, as predicted by Eq.(1) in the main text. In our case, since the bond resistance does not change with the gap in a jump-wise manner but rather as a continuous exponential, the resistance noise does not diverge at threshold and the singularity is washed out[2].

Molecular Dynamics-Langevin method (MD-L) for memristors

Some of our preliminary two-dimensional molecular dynamics simulations[3] showed filament-like oxygen vacancy cluster formation and a certain degree of orientation that resemble experimental results obtained in TiO_2 memristors. We have shown recently[4] that Joule heating causes current-controlled negative differential resistance (CC-NDR) in TiO_{2-x} by constructing an analytical model of the voltage-current V=V(I) characteristic based on polaronic transport for conductivity and Newton's Law of Cooling and comparing it to the data at hand. This threshold switching is the soft breakdown observed during electroforming of TiO_{2-x} and other transition-metal-oxide based memristors, as well as a precursor to ON or SET switching of unipolar memristors from their high to their low resistance states. Here, we combine our MD simulations with Newton's Law of cooling in the bottleneck in order to model the noise in the memristors.

We have developed a MD-Langevin equation for diffusion of ionic species under an external force F acting on ions at positions r_i, subject to their interaction $W(r_i$-$r_j)$ and the pinning/crystalline potential $U(\{r_i\})$ (see Supplementary Ref. 5 and references therein):

$$\eta\frac{dr_i^\alpha}{dt} = F^\alpha(t) - \sum_{j\neq i}\frac{\partial W(r_i-r_j)}{\partial r_i^\alpha} - \frac{\partial U(\{r_i\})}{\partial r_i^\alpha} + \sqrt{2k_BT\eta}\,\xi_i^\alpha(t), \tag{3}$$

where the friction η is related to the diffusion coefficient as $D = k_BT/\eta$ and the random forces satisfy fluctuation-dissipation relation $\langle \xi_i^\alpha(0)\xi_j^\beta(\tau)\rangle = \delta(\tau)\delta_{\alpha\beta}\delta_{ij}$, where $\alpha = x, y, z$ is the

Cartesian index[3]. In the dynamical equation, the friction force $\eta \frac{dr_i^\alpha}{dt}$ with the viscosity constant η is balanced by the electric force F of the applied voltage pulse, the vacancy-vacancy interaction defined by the potential W, the interaction of a vacancy with the potential U (defect pinning or crystal field) and the thermal noise ξ.

The V_O^{++} vacancy-vacancy interaction, which is crucial for understanding vacancy clustering and memristor switching, can be modeled as in Supplementary Ref. 6. It has been approximated in the present work as a Lennard-Jones (LJ) potential with Coulomb repulsion [4]:

$$W(r) = E_{LJ}\left(\frac{r_m^{12}}{r^{12}} - 2\frac{r_m^6}{r^6}\right) + E_c\frac{r_m}{r}, \qquad (4)$$

where E_{LJ} is the LJ energy, i.e. the depth of the LJ (first term) of the potential at $r = r_m$, $E_c = \frac{e^2}{\pi\epsilon_0\epsilon r_m}$ is the Coulomb energy, $r_m = 0.125$nm, and $E_c/E_{LJ} = 1/2$. This results in the positions of the potential maxima at $r_{max} \approx 2r_m$ and the height of the potential barrier on the order of the depth of the potential well. Consistent with the size of our measured memristors, the size of the simulation box was $20r_m$ between the top and bottom terminals, and we use periodic boundary conditions along the memristor film with a period of $10r_m$. The characteristic time scale is defined by the Langevin friction η and LJ energy: $\tau = \frac{\eta\, r_m^2}{E_{LJ}}$. In our simulations, we have used the stochastic Euler-Maruyama method [5] with the parameters $A\Delta = 8\tau E_{LJ}/r_m$, where the voltage pulse amplitude is A and the pulse duration is τ. The time step was 0.005τ.

As we have shown previously[3], the vacancies tend to form filament-like clusters after the voltage pulse even in the absence of a pinning potential U. These filamentary clusters can serve as current conducting paths between electrodes. They can be destroyed[3] by another electric pulse, which allows memristors to switch between low and high resistive states. Here, we have included the potential U created by immobile charged inclusions (shown as green circles in the atomistic figures):

$$U = \sum E_c^{pin}\frac{r_m}{r} \qquad (5)$$

with $E_c^{pin}/E_c = 10$. Our simulations showed that pinning promotes the formation of controllable bridges between memristor terminals, in agreement with experiments on devices with columnar

defects. An example of the evolution of the vacancy distribution within the described model is shown in Supplementary Fig. 3. The present model is fully generic and will result in similar behavior for other systems with mobile ion monospecies interacting via a potential like (eq. 4), i.e. cations, anions, etc. The main feature of the potential (eq. 4) is that it corresponds to an attractive interaction of nearest neighbors while more distant species repel each other via a screened Coulomb potential. The model reveals a strong enhancement of noise in the regime close to QPC formation, where the system randomly ends up in either connected (metallic) or disconnected (insulating) states, Supplementary Fig. 5.

The model also explains other behavior in our devices. Except for the peaks at the quantized conductance values, the measured noise in our TaOx samples varied slowly with conductance. Each peak in the noise vs. conductance plot could be caused by a localized hot spot in one or more conducting channels. The local hot spot heating hypothesis can be tested by analyzing the noise at lower ambient temperatures, as shown in Fig. 3 in the main text and Supplementary Fig. 2. Importantly, the 174 K data show low sensitivity of the noise to ambient temperature in the regime near G_Q and in the tunneling regime at $G < G_Q$. In the Ohmic regime $G > G_Q$, we see that the noise diminishes at lower temperatures. The absence of an ambient temperature effect near the single conductance quantum implies that the noise is chiefly determined by the local heating at the few-atom bottleneck. This is further evidenced by the statistics of breaking-making contact as a function of vacancy concentration n (Supplementary Fig. 4). The probability at the critical vacancy content n_c for making-breaking the contact is about 50%, and the standard deviation of the conductance G is maximum, which is corroborated by the experimental data shown in Figure 2 in the main text.

In order to further analyse the noise characteristics, we have extended the simulations into a regime after a point contact has formed. We assumed that almost all the electric power was dissipated in the bottleneck region, producing local heating that affected only a few vacancies. Local heating to the temperature T_b of the bottleneck can be described by Newton's cooling law:

$$\frac{dT_b}{dt} = \frac{V_{loc}^2}{CR(t)} - \lambda(T_b - T_0), \qquad (6)$$

where $V_{loc}^2/R(t)$ is the Joule heat dissipated by the fluctuating bottleneck resistance $R(t)$, subject to the local voltage bias at the bottleneck V_{loc}, heating it to the local temperature T_b, with T_0 being the ambient temperature, C the specific heat, and λ the thermal resistance coefficient at the bottleneck.

We model the bottleneck with four particles (instead of the single fluctuator used above to understand the noise peaks) located at the coordinates $\mathbf{x}_1$,$\mathbf{x}_2$,$\mathbf{x}_3$,$\mathbf{x}_4$, where the minimum distances between the first two particles $\mathbf{x}_1$,$\mathbf{x}_2$ and the second two $\mathbf{x}_3$,$\mathbf{x}_4$ were associated with the gap $\Delta=\min(|\mathbf{x}_i-\mathbf{x}_j|)$ (with i=1,2 and j=3,4) between two large clusters of particles attached to the top and bottom electrodes, Supplementary Fig.5a. We assumed that the resistance of the memristors is dominated by the bottleneck resistance $R(t)$, which exponentially increases with the gap Δ,

$$R(t) = R_Q \exp([\Delta(t) - r_m]/d), \tag{7}$$

with a characteristic electron tunneling length d, where the resistance is R_Q (i.e. $1/G_Q$) when Δ is the equilibrium distance r_m. Thus, this system fluctuates between instantaneous conductance values of G_Q and a much smaller tunneling conductance, so the time average conductance $\langle G \rangle$ will be very roughly $0.5G_Q$. The simplest (and realistic) case is when the characteristic temperature relaxation time $1/\lambda$ is much shorter than the time scale of vacancy diffusion, $1/\lambda \ll \tau$, and the local temperature is much higher than the ambient temperature. Therefore, the temperature always reaches its stationary value for a given vacancy configuration:

$$T_{b0} = T_0 + \frac{V_{loc}^2}{\lambda CR}, \tag{8}$$

while the vacancies outside the bottleneck remain at the ambient T_0, which is neglected compared to the bottleneck temperature. We further assumed that $T_{b0} - T_0 \approx 0.2E_c$, where E_c is the characteristic Coulomb energy. Therefore, when performing MD-L simulations, we assume that the local heating to T_{b0} affects only the four marked vacancies in the bottleneck in Supplementary Fig. 5a and ignore random forces on all other vacancies. The temperature is controlled by the resistance R, Eq. (8), which depends on the gap in the bottleneck, Eq. (7), in a self-consistent manner. At a constant temperature, the electronic noise spectra of the gap in the

bottleneck should have a $1/f^2$ dependence, since the gap is a relative distance between the vacancies that are modelled as Brownian particles[1]. However, the self-consistent fluctuations of the bottleneck temperature modulated by the exponential dependence on the gap result in filtering of the spectra, which then exhibits a crossover from $1/f$ noise seen at lower f to a $1/f^2$ dependence at higher frequencies (Supplementary Fig. 5c) and seen in the experimental data in Fig. 4 in the main text.

Supplementary References

1. Kogan, Sh. *Electronic Noise and Fluctuations in Solids* (Cambridge Univ. Press, Cambridge, 1996).
2. Beloborodov, I.S., Lopatin, A.V., Vinokur, V.M. & Efetov, K.B. Granular electronic systems, *Rev. Mod. Phys.* **79**, 469-517 (2007).
3. Savel'ev, S.E., Alexandrov, A.S., Bratkovsky, A.M. & Williams, R.S. Molecular dynamics simulations of oxide memory resistors (memristors), *Nanotechnology* **22**, 254011 (2011).
4. Alexandrov, A.S., Bratkovsky, A.M., Bridle,B., Savel'ev, S.E., Strukov, D.B. &. Williams, R.S. Current-controlled negative differential resistance due to Joule heating in TiO2, *Appl. Phys. Lett*. **99**, 202104 (2011).
5. Savel'ev, S.E., Marchesoni, F., & Nori, F. Stochastic transport of interacting particles in periodically driven ratchets, *Phys. Rev. E* **70**, 061107 (2004).
6. Meis, C. & Fleche, J.L. Study of the solubility limit of oxygen vacancies in TiO_{2-x} using molecular dynamics, *Solid State Ionics* **101**, 333-335 (1997).
